# Environmental DNA as a ‘Snapshot’ of Fish Distribution: A Case Study of Japanese Jack Mackerel in Maizuru Bay, Sea of Japan

**DOI:** 10.1371/journal.pone.0149786

**Published:** 2016-03-02

**Authors:** Satoshi Yamamoto, Kenji Minami, Keiichi Fukaya, Kohji Takahashi, Hideki Sawada, Hiroaki Murakami, Satsuki Tsuji, Hiroki Hashizume, Shou Kubonaga, Tomoya Horiuchi, Masamichi Hongo, Jo Nishida, Yuta Okugawa, Ayaka Fujiwara, Miho Fukuda, Shunsuke Hidaka, Keita W. Suzuki, Masaki Miya, Hitoshi Araki, Hiroki Yamanaka, Atsushi Maruyama, Kazushi Miyashita, Reiji Masuda, Toshifumi Minamoto, Michio Kondoh

**Affiliations:** 1 Graduate School of Human Development and Environment, Kobe University, Hyogo, Japan; 2 Faculty of Fisheries Sciences, Hokkaido University, Hokkaido, Japan; 3 The Institute of Statistical Mathematics, Tokyo, Japan; 4 Maizuru Fisheries Research Station, Kyoto University, Kyoto, Japan; 5 Department of Environmental Solution Technology, Faculty of Science and Technology, Ryukoku University, Shiga, Japan; 6 Graduate School of Environmental Science, Hokkaido University, Hokkaido, Japan; 7 Faculty of Human Development, Kobe University, Hyogo, Japan; 8 Natural History Museum & Institute, Chiba, Chiba, Japan; 9 Research Faculty of Agriculture, Hokkaido University, Hokkaido, Japan; 10 Field Science Center for the Northern Biosphere, Hokkaido University, Hokkaido, Japan; Central Michigan University, UNITED STATES

## Abstract

Recent studies in streams and ponds have demonstrated that the distribution and biomass of aquatic organisms can be estimated by detection and quantification of environmental DNA (eDNA). In more open systems such as seas, it is not evident whether eDNA can represent the distribution and biomass of aquatic organisms because various environmental factors (e.g., water flow) are expected to affect eDNA distribution and concentration. To test the relationships between the distribution of fish and eDNA, we conducted a grid survey in Maizuru Bay, Sea of Japan, and sampled surface and bottom waters while monitoring biomass of the Japanese jack mackerel (*Trachurus japonicus*) using echo sounder technology. A linear model showed a high *R*^2^ value (0.665) without outlier data points, and the association between estimated eDNA concentrations from the surface water samples and echo intensity was significantly positive, suggesting that the estimated spatial variation in eDNA concentration can reflect the local biomass of the jack mackerel. We also found that a best-fit model included echo intensity obtained within 10–150 m from water sampling sites, indicating that the estimated eDNA concentration most likely reflects fish biomass within 150 m in the bay. Although eDNA from a wholesale fish market partially affected eDNA concentration, we conclude that eDNA generally provides a ‘snapshot’ of fish distribution and biomass in a large area. Further studies in which dynamics of eDNA under field conditions (e.g., patterns of release, degradation, and diffusion of eDNA) are taken into account will provide a better estimate of fish distribution and biomass based on eDNA.

## Introduction

Surveillance of fish species composition and biomass provides useful information for management and conservation of marine and freshwater ecosystems. For example, a single survey of fish composition at a local habitat may suggest interactions among species (e.g., co-occurrence patterns) [1,2], while a time-series of surveys may reveal temporal patterns of fish migration [3,4]. In addition, long-term monitoring can reveal time lags between invasion and explosive population growth of alien fish [5], increases in invasive fish abundance related to decreases in population sizes of native fish species [6], and effects of climatic fluctuations on population growth of estuarine fish species [7]. Furthermore, comparing the composition and biomass of fish communities between habitats can reveal important environmental requirements for the survival of young fish [8].

Various sampling methods have been used for fish surveillance, depending on the objectives of studies and the field conditions; each has advantages and disadvantages. For example, echo sounder, which generates an acoustic pulse and detects echos from fish, allows one to survey large geographical areas in a short period of time, but it is inadequate for species identification and surveillance in environments with many obstacles such as coral reefs. In such environments, underwater visual censuses should be effective for fish counts and species identification, although data from these methods may contain bias resulting from fish and diver (observer) behavior [9–12]. Moreover, underwater visual censuses require more manpower and/or longer investigation times for large areas than methods using echo sounder. Surveys using various types of fishing nets have also been conducted. Although the numbers of fish species recovered by surveys with beach seines and bottom trawls are similar to those from underwater visual censuses [13], these surveys stress fish individuals and therefore are not appropriate for threatened species.

Detection and quantification of species-specific environmental DNA (eDNA), DNA molecules originally shed from the bodies of organisms in the environment (e.g., [14]), might eliminate the drawbacks of the aforementioned methods. Sampling of eDNA requires only small amounts of water (e.g., 1 L or less [13,15,16]), and species-specific eDNA can be detected and quantified by quantitative PCR using species-specific primers and probes. Thus, the eDNA method has species-specific sensitivity and requires less field time than the other survey methods. The method was first developed to detect a frog species [17], and other endangered and rare aquatic species [18–21] as well as invasive species [15,22] have been detected using this method. Moreover, not only does this method have higher detection rates than traditional methods [13,19], but a correlation between eDNA quantity and species biomass has also been found in both experimental [23] and field studies [18,19,24]. Hence, using the eDNA method, the transient distribution of a specific species over a large area (e.g., bays and large lakes) can be identified. Such snapshots of species distribution would be useful for the management and monitoring of fish in seas and large lakes. However, the movement of eDNA in seas is complex, for instance, current might transfer eDNA intricately. Therefore, it is uncertain whether the spatial variation in the quantity of eDNA reflects the local biomass and abundance of species.

**Fig 1:**
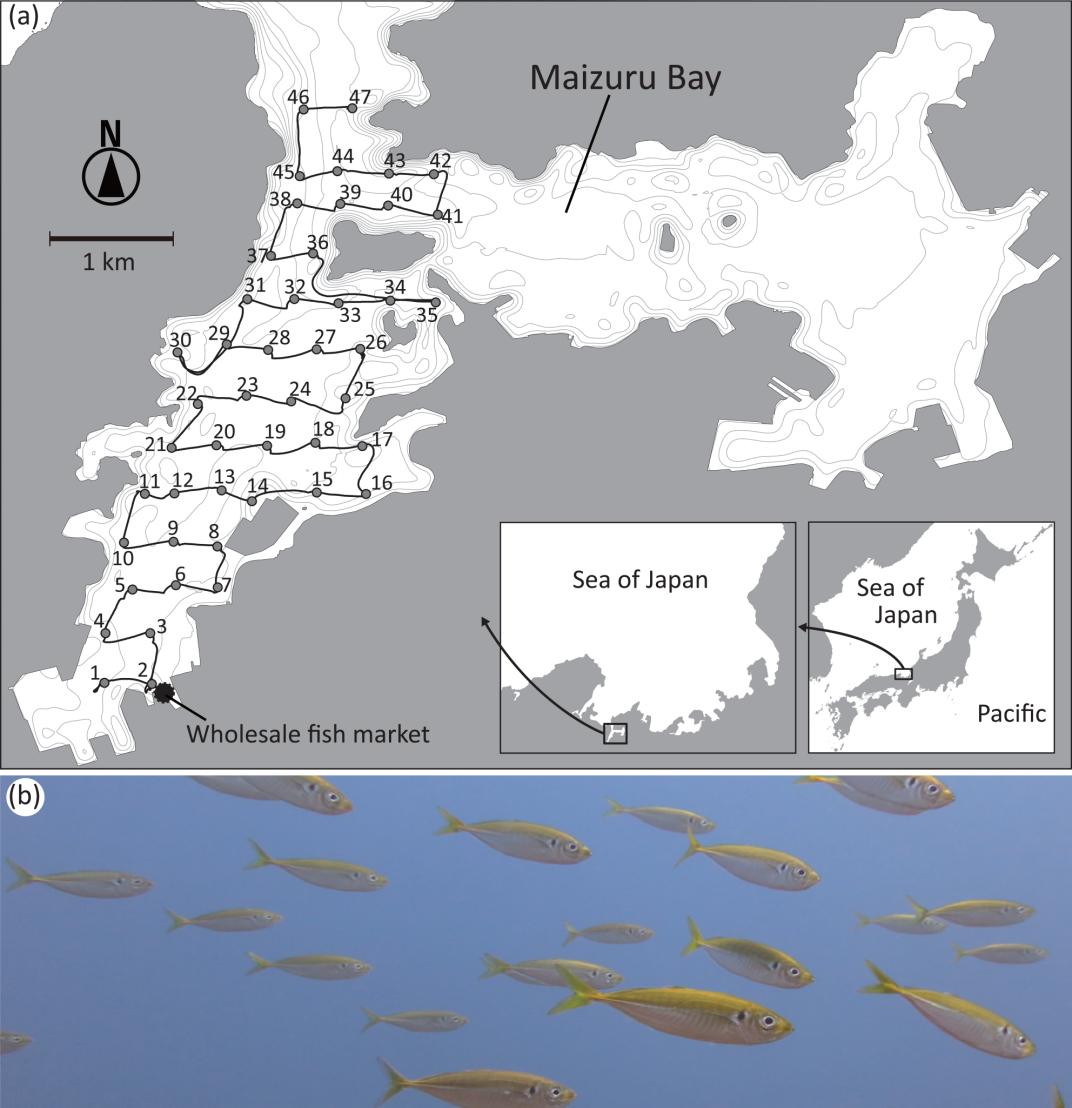
Research site and target species. Location of Maizuru Bay, sampling stations and cruise track in Maizuru Bay (a) and a picture of the target species, jack mackerel (b). Gray areas indicate land masses and gray lines indicate depth contours with an interval of 4 m.

In the present study, we assessed whether the distribution of a fish species in a sea could be monitored based on the spatial variation in the quantity of eDNA. To this end, we collected seawater samples over a wide area in Maizuru Bay, Sea of Japan, and quantified eDNA of the Japanese jack mackerel (*Trachurus japonicus*) (Fig 1). This bay is semi-closed body of water located along the temperate Sea of Japan and with a tidal range of only ~50 cm and a weak current (5–20 cm s^-1^) [25], providing an ideal site to evaluate the efficacy of eDNA surveys. The jack mackerel was targeted because our previous investigations by underwater visual census revealed that this commercially important fish is numerically the most dominant in the area from spring to autumn (see Materials and Methods) [26–28]. We conducted an acoustic survey to estimate fish biomass simultaneously with water collection. Based on the two different data sets, we examined whether the eDNA concentration of the jack mackerel varied spatially in the bay, and whether the spatial variation of eDNA concentration was correlated to the biomass estimated by the acoustic survey. Our goal was to evaluate the applicability and limitations of the eDNA method for revealing species distribution and biomass in a large aquatic area.

## Materials and Methods

### Ethics statement

Field research in the present study was approved by the harbormaster of Maizuru Bay (Permission Number 191 issued at 9 June 2014).

### Research site and field survey

To examine the association between the estimated concentration of jack mackerel eDNA and the biomass estimation using echo sounder, water sampling and an acoustic survey were conducted on 18 June 2014 in west Maizuru Bay, which has a surface area of ca. 11 km^2^ (35.481°N, 135. 332°E; Fig 1). Echo sounder survey cannot discriminate fish species clearly. Therefore, jack mackerel is the most suitable fish for our research purpose because of its abundance. Underwater visual censuses have revealed that abundance of jack mackerel is much larger than any other fishes in Maizuru Bay [26]. For example, the total abundance of jack mackerel during census period (approximately two weekly intervals between 1 January 2002 and 21 December 2006) was more than 21,000 while the second most abundant fish, Japanese anchovy (*Engraulis japonicus*), was ~8,000. Moreover, the underwater visual censuses indicated that jack mackerel usually exceeds 200 individuals/400m^2^ in June in Maizuru Bay while only four species reach 20–150 individuals/400m^2^ even in years that each species was abundant. In addition to those species, the other species are 20 or less individuals/400m^2^. Therefore, we can assume that detected signals by echo sounder in June in Maizuru Bay predominantly indicate jack mackerel. Only Japanese anchovy occasionally shows ~200 individuals/400m^2^. However, because the average body size of Japanese anchovy is ~2 cm in our research season, echo intensity of Japanese anchovy individual would be extremely small (e.g., less than 1% of echo intensity of small jack mackerel individuals) [29]. Therefore, echo signals from the anchovy scarcely contribute to our analyses. Thus, we chose jack mackerel as a target fish and Maizuru Bay as research site in the present study.

The echo sounder surveys started from the mouth of the bay (near St. 47) and moved southwestwardly to the end of the bay (near St. 1). Our survey cruises were conducted during the day, and it took 6 hours. The tracks of our ship are depicted in Fig 1. There is a single wholesale fish market at the southwest corner of the bay (Fig 1), which we considered a potential source of eDNA and hence was included in our modeling.

### Water sampling, eDNA extraction from filter, quantitative PCR

Seawater samples for eDNA analyses were collected both from the sea surface using buckets and from ~1.5 m above the sea bottom using van Dorn water samplers at 47 sites in west Maizuru Bay on 18 June 2014 (Fig 1). We collected 3 L or more of seawater by one cast of sampling devices from both the surface and bottom at each sampling site. Then, three 1 L samples were subsampled using measuring cup (i.e., the three filters were subsets of a single water collection). After collecting the water samples, we immediately filtered 1 L through a 47 mm diameter glass microfiber filter (nominal pore size 0.7 μm; GE Healthcare Life Science [Whatman]) on board. To lower the level of cross-contamination, buckets were washed twice or more with surface water at each sampling site and van Dorn samplers were put in bottom water for several minutes before each water collection. Although we conducted washing of water-sampling devices rather than bleaching to reduce on-board operation, this procedure should successfully prevent carryover of eDNA between sampling stations (S1 Text). On the other hand, filtering devices (i.e., filter funnels and measuring cups used for filtration) were bleached after every filtration using 0.1% sodium hypochlorite. In addition, to verify the effectiveness of the bleaching, we filtered artificial seawater with a randomly selected filter funnel and measuring cup at every fifth site (equipment negative control). The filters were placed in a freezer immediately after filtration until eDNA extraction.

Total eDNA was extracted from each filter using a DNeasy Blood and Tissue Kit (Qiagen, Hilden, Germany) with a minor modification to adjust for eDNA extraction. Briefly, a sample filter was placed in the suspended part of a Salivette tube (Sarstedt, Nümbrecht, Germany). Then, 440 μL solution composed of 40 μL Proteinase K and 400 μL AL Buffer was put on the filter and the tube was incubated at 56°C for 30 min. After incubation, the liquid held in the filter was collected by centrifugation. To increase the yield of eDNA, 200 μL TE buffer was put on the filter and the liquid was again gathered by centrifugation. We added 200 μL AL buffer and 600 μL ethanol to the collected liquid, and transferred the mixture to a spin column. Subsequently, we followed the manufacturer’s instructions and total eDNA was eluted in 100 μL AE buffer. To check for cross-contamination during eDNA extraction procedures, we simultaneously extracted eDNA from an unused filter (extraction negative control). We first extracted eDNA from one of three filters from each of 94 sampling points (both surface and bottom sampling at 47 sites). The set of filters was defined as filter series 1. We then extracted eDNA from one of the other filters and the set of filters was defined as filter series 2. Finally, the remaining filters were DNA-extracted and defined as filter series 3.

To evaluate the amount of eDNA derived from jack mackerel, quantification of the copy of mitochondrial cytochrome *b* (CytB) gene was performed using real-time TaqMan^®^ PCR with a StepOne-Plus™ Real-Time PCR system (Life Technologies, Foster City, USA). We amplified and quantified eDNA using primers and a probe specific to jack mackerel: forward primer, 5´-CAG ATA TCG CAA CCG CCT TT-3´; reverse primer, 5´-CCG ATG TGA AGG TAA ATG CAA A-3´; probe, 5´-FAM-TAT GCA CGC CAA CGG CGC CT-TAMRA-3´ (Minamoto *et al*., unpublished data). The primers specifically amplify a 127 bp fragment of the jack mackerel CytB gene. Each 20 μL TaqMan reaction contained 2 μL extracted eDNA solution, a final concentration of 900 nM forward and reverse primers and 125 nM TaqMan probe in 1 × PCR master mix (TaqMan gene expression master mix). Quantitative PCR (qPCR) was performed with the following conditions: 2 min at 50°C, 10 min at 95°C, and 55 cycles of 15 s at 95°C and 1 min at 60°C. For each eDNA sample (i.e., filter), PCR was performed in triplicate. To quantify the number of jack mackerel CytB genes in each 2 μL eDNA solution sample, we simultaneously performed qPCR in triplicate using a dilution series of standards containing 3 × 10^1^–3 × 10^4^ copies of a commercially synthesized artificial DNA fragment that included the jack mackerel CytB sequence amplifiable with the above primer set. The artificial DNA fragments were pUC57 plasmids containing 327 bp of the partial CytB gene: the amplified 127 bp region and 100 bp upstream and downstream of the amplified region. Prior to qPCR analysis, the plasmids were digested with a restriction enzyme (EcoRI). In addition, a 2 μL pure water sample was analyzed simultaneously in triplicate as a negative control in the PCR (PCR negative control). In all the runs, *R*^2^ values of calibration curves were more than 0.992, the range of slopes were between –3.951 and –3.370, and the range of intercepts were between 39.055 and 42.264. Based on the calibration curve of each run and the Ct value of each sample, the copy number of CytB gene fragment was calculated. Finally, to confirm the species-specificity of those primers and the probe, we determined sequences of 47 samples that were randomly selected from amplified samples by Sanger sequencing. As a result of BLAST search using NCBI nucleotide database, all the sequenced fragments had a jack mackerel CytB gene sequence.

### Acoustic survey of fish biomass using echo sounder

Jack mackerel biomass was estimated using a calibrated quantitative echo sounder following a standard acoustic survey method (e.g., [30]). We used the echo sounder, KCE300 (Sonic Co. Ltd., Tokyo, Japan), with a T-182 transducer (frequency, 120 kHz; beam type, split-beam; beam width, 8.5°; pulse duration, 0.6 ms; ping rate, 0.2 s). The transducer was mounted off the side of the research vessel at a depth of ~1.0 m to avoid cavitation bubbles generated by the research vessel. The acoustic devices were operated during the entire survey cruise (Fig 1) and all signals were recorded. The average ship speed was ~4 knots between sampling stations, although the ship slowed when approaching a sampling site and completely stopped to collect water samples.

We eliminated noise from the obtained echo intensity data using Echoview ver. 6.0 (Echoview software Pty. Ltd., Tasmania, Australia). According to a regression described in a previous study [31], we assumed that the echo intensity of a jack mackerel individual of 3 cm SL was –59.6 dB. We set the threshold size at 3 cm SL considering the size range of jack mackerels found in this area; a mackerel of ca. 3 cm SL should be the minimum size during the investigated season in Maizuru Bay [28] and those greater than 3 cm SL showed more intensive signals. Therefore, we eliminated signals less than –59.6 dB. Signals between the sea bottom and 0.5 m above the sea bottom were also eliminated to avoid possible confounding with the acoustic dead zone, which was calculated based on pulse length and local bathymetry [32]. Finally, we eliminated the signals of bubbles generated by the movement of the screw propeller. After eliminating these noises, we re-obtained echo intensity data to assess the association with eDNA concentration (see below). Note that the obtained echo intensity data would include echo signals from a variety of fish rather than jack mackerel alone. However, we can assume the obtained echo intensity as a biomass index of jack mackerel as explained in the above section (e.g., predominance of jack mackerel in Maizuru Bay and negligible contribution of Japanese anchovy to echo intensity data).

### Data analysis

Because we did not have any *a priori* knowledge of the relevant spatial scale of fish biomass reflecting the spatial pattern of eDNA concentration inside the bay, we obtained 20 datasets of local echo intensity, which was determined as the cumulative echo signals within a defined volume of the water column, from the original echo intensity data (S1 Fig). Note that original echo intensity data have volume backscattering coefficient that indicates echo strength per a 1-m^3^ water cube (hereafter, s_v_) on cruise trajectories. To estimate the local echo intensity, we considered five levels of horizontal range (buffer area) and four levels of vertical range, which were used to define the water columns surrounding each sampling station. Horizontal ranges were within a 10, 30, 50, 150 and 250 m radius from each sampling station (hereafter, each horizontal range is called as buffer, e.g., “10 m buffer”), and vertical ranges were within 2, 5, and 10 m from both the surface and bottom at each sampling station, as well as the entire vertical range of the sea (i.e., between the transducer and 0.5 m above sea bottom). All possible combinations of these horizontal and vertical ranges yielded 20 datasets of local echo intensity for each sampling station: for example, a station might have a local echo intensity value of a 10 m buffer/2 m vertical water column, a 10 m buffer/5 m vertical water column, and so on. Within each water column, we obtained a series of s_v_ values and the integrated s_v_ between the vertical ranges (e.g., within 2 m from both the surface and bottom), along the cruise trajectory. This integrated s_v_ value is identical with area backscattering coefficient (hereafter, s_a_), which indicates echo strength per a water column with a cross-sectional area of 1 m^2^ (i.e., s_a_ correlates a fish biomass just below our research vessel). Then the average of integrated s_v_ values within each water column was multiplied by the area of the water column, yielding the estimated cumulative echo signals within each column (we note that the area of land was adequately considered in the calculation of the cumulative echo signal; S1 Fig; see S1 Table for data of local echo intensity). Finally, we obtained the local echo intensity for each water column as the logarithm of cumulative echo signals.

We analyzed the relationship between echo intensity and the estimated eDNA concentration using the linear regression method along with the variable selection procedure with information criteria. We considered eDNA concentration, which is the estimated copy number of eDNA in each PCR replicate rather than the average value of PCR replicates, as the dependent variable. Explanatory variables included: (1) local echo intensity (represented as ‘echo’ in the following formula), as our primary interest; (2) vertical position of water samples (i.e., surface or bottom, represented as ‘depth’); (3) filter series (‘filter’); and (4) a measure of the inverse of the distance of each sampling station from the fish market (defined as the exponential of the negative of the geographic distance in km between the fish market and each sampling station, represented as ‘inv-dist’) as covariates. As mentioned above, there were 20 values of local echo intensity (echo) for each sampling site corresponding to the differences in the horizontal and vertical spatial scale of the water column. We assumed that the spatial scale of the local echo intensity was constant among sampling sites, whereas it might vary across the vertical sampling position. The three covariates appeared to affect the observed variation in eDNA concentrations. Filter series was considered because DNA concentration seems to decrease in later filter series; DNA extractions were conducted from series 1 to 3 at different times and filter series 2 and 3 would probably undergo freezing and thawing, causing reductions in eDNA concentration (S1 Table). Distance from the fish market was especially likely to affect the spatial eDNA pattern because various fish species, including jack mackerel, are processed there. In fact, a preliminary MiFish metabarcoding analysis [33] of the sampled water detected mitochondrial DNA of fish species that live outside the bay but are processed in the fish market (e.g., flying fishes of the genus *Cypselurus*) in water sampled at St. 2, the nearest site to the fish market (unpublished data). In addition, we also considered (5) a binary (i.e., dummy) explanatory variable that indicates whether the local echo intensity was zero or not (represented as ‘no-echo’), because in some water columns the cumulative echo signal was zero-valued and hence we could not take the logarithm. Then we assigned echo = 0 and no-echo = 1 for these data for use in the analysis. Note that an explanatory variable, no-echo, was only included in the regression models that involved its counterpart variable, echo. As for echo, interactions between no-echo and other covariates were also considered. No serious collinearity was found in these explanatory variables (1–5), which was confirmed by calculating the variance inflation factor (S2 Table). The resulting full model formula (shown in the conventional expression in R language) was: eDNA ~ inv-dist + depth + filter + inv-dist:depth + inv-dist:filter + depth:filter + inv-dist:depth:filter + echo + no-echo + depth:echo + depth:no-echo + filter:echo + filter:no-echo + depth:filter:echo + depth:filter:no-echo, where x:y indicates an interaction term of x and y. We assumed that echo intensity and distance from the fish market did not interact with each other. We used the Akaike Information Criterion (AIC) to determine the best set of predictor variables as well as the best spatial scale of water columns. The above analyses were conducted for two different sets of data: the data from all 47 sites × 2 depths, and the data without outliers, where samples showing extremely high eDNA concentrations (S1 Table) were excluded. Analyses were performed using R version 3.1.3 [34]. The data matrix and the R script used for regression analyses are described in Supplementary Materials (S1 File).

## Results

### Spatial variation in jack mackerel eDNA and echo intensity

**Fig 2:**
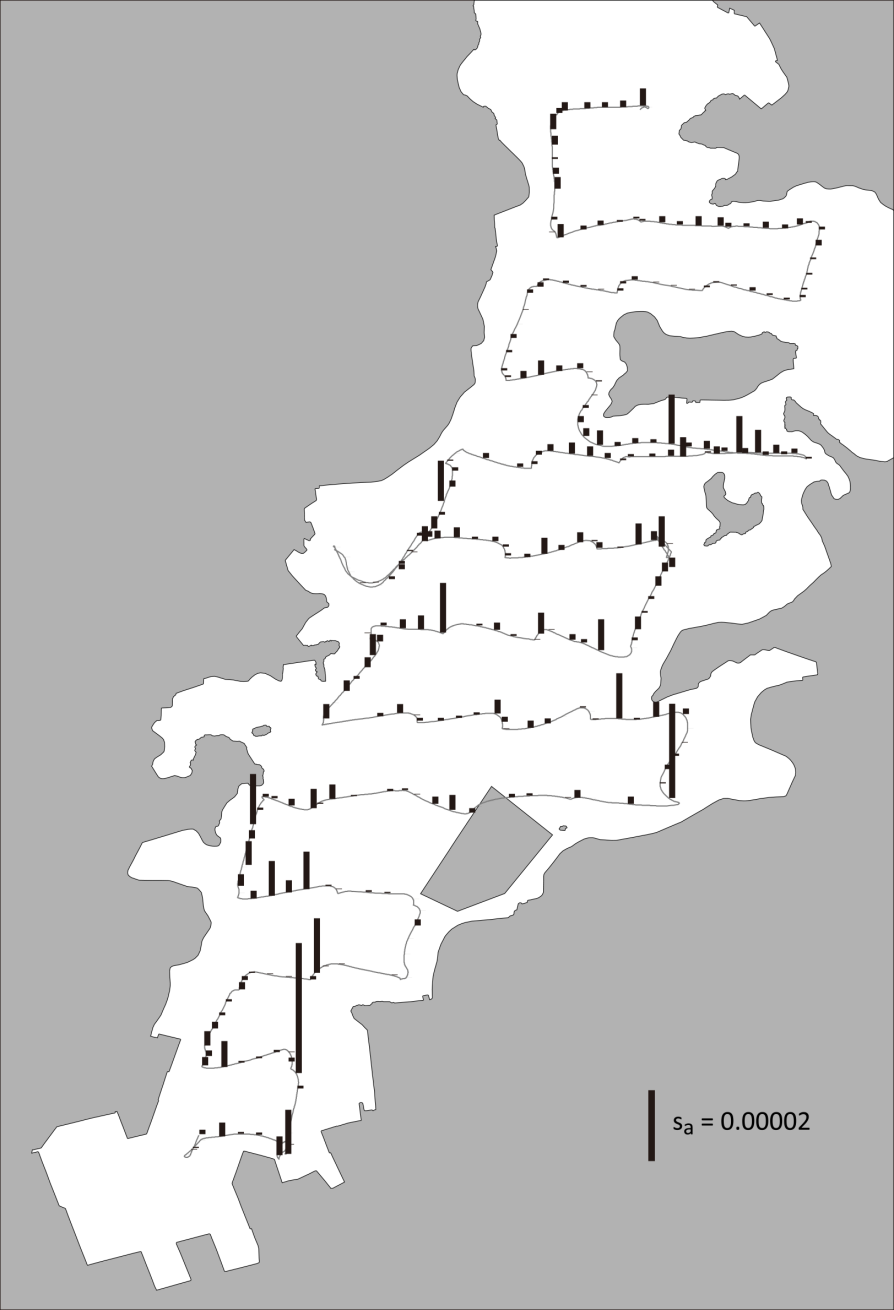
Spatial approximation of jack mackerel eDNA concentration. Based on CytB gene copy number in a 2 μL template DNA solution at the 47 sampling station, spatial variation of jack mackerel eDNA in west Maizuru Bay was estimated by approximation. The level of the approximate eDNA concentration is indicated by colors between red (relatively high concentration) and blue (low concentration). White areas suggest that the concentration approximated using a regularized spline is ≤ 0. Spatial approximation was performed using a regularized spline with a tension parameter of 40.

The three filter replicates from each sampling event resulted in different eDNA concentrations; filter series 1 generally had higher concentrations than those from the other two (S1 Table). However, the trends of eDNA concentration among filters from the same series were similar among filter series (*r* = 0.87, 0.84 and 0.86 for filter series 1 vs. 2, 1 vs. 3 and 2 vs. 3, respectively; *p* < 0.0001 for all pairs). The quantity of jack mackerel eDNA varied among the 47 sampling sites (Fig 2; S1 Table). The highest concentrations were detected from both the surface and bottom waters at St. 2, being two to five times higher than the second highest eDNA concentrations of each filter series. In addition, bottom water from St. 27 had an eDNA concentration approximately three times higher than filters with the third highest eDNA concentrations. Jack mackerel eDNA was detected in the surface water of almost all of the sites but there were some sites where it was not detected in the bottom water. All the negative controls (i.e., equipment, extraction and PCR negative controls) were not PCR-amplified, indicating that there was no carryover through the filtration device and no cross-contamination among samples during molecular experiments.

**Fig 3:**
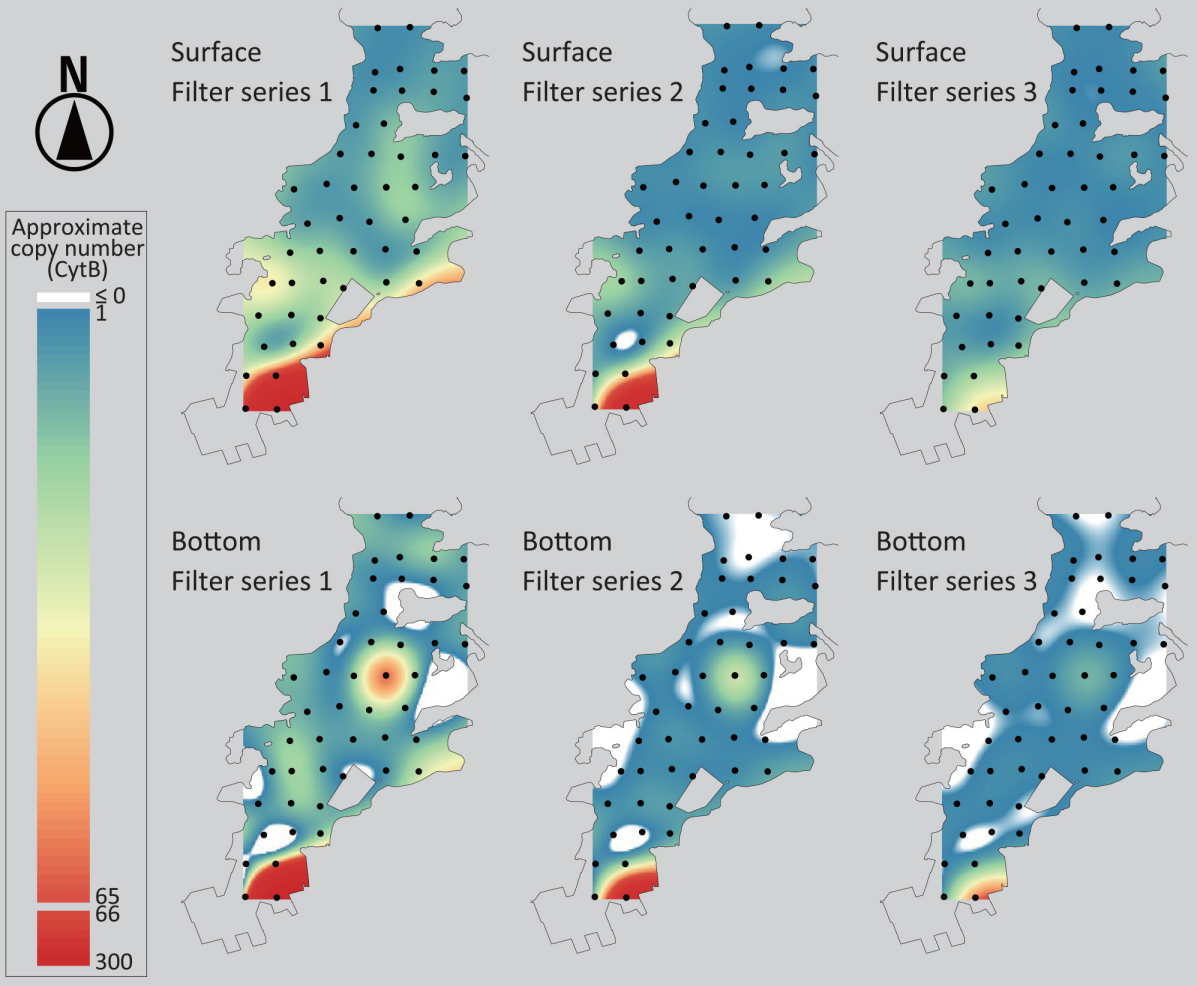
Observed fish biomass using echo sounder. Vertical bar on the cruise track (gray line) indicates local s_a_ values (i.e., fish biomass observed using quantitative echo sounder), which is the integrated s_v_ of a water column with a cross-sectional area of 1 m^2^. This figure is depicted according to s_a_ extracted every 80-m intervals. Note that this figure shows a summary of field observation using echo sounder. We used s_v_ values rather than s_a_ values as index of fish biomass in regression analyses (see S1 Fig).

**Fig 4:**
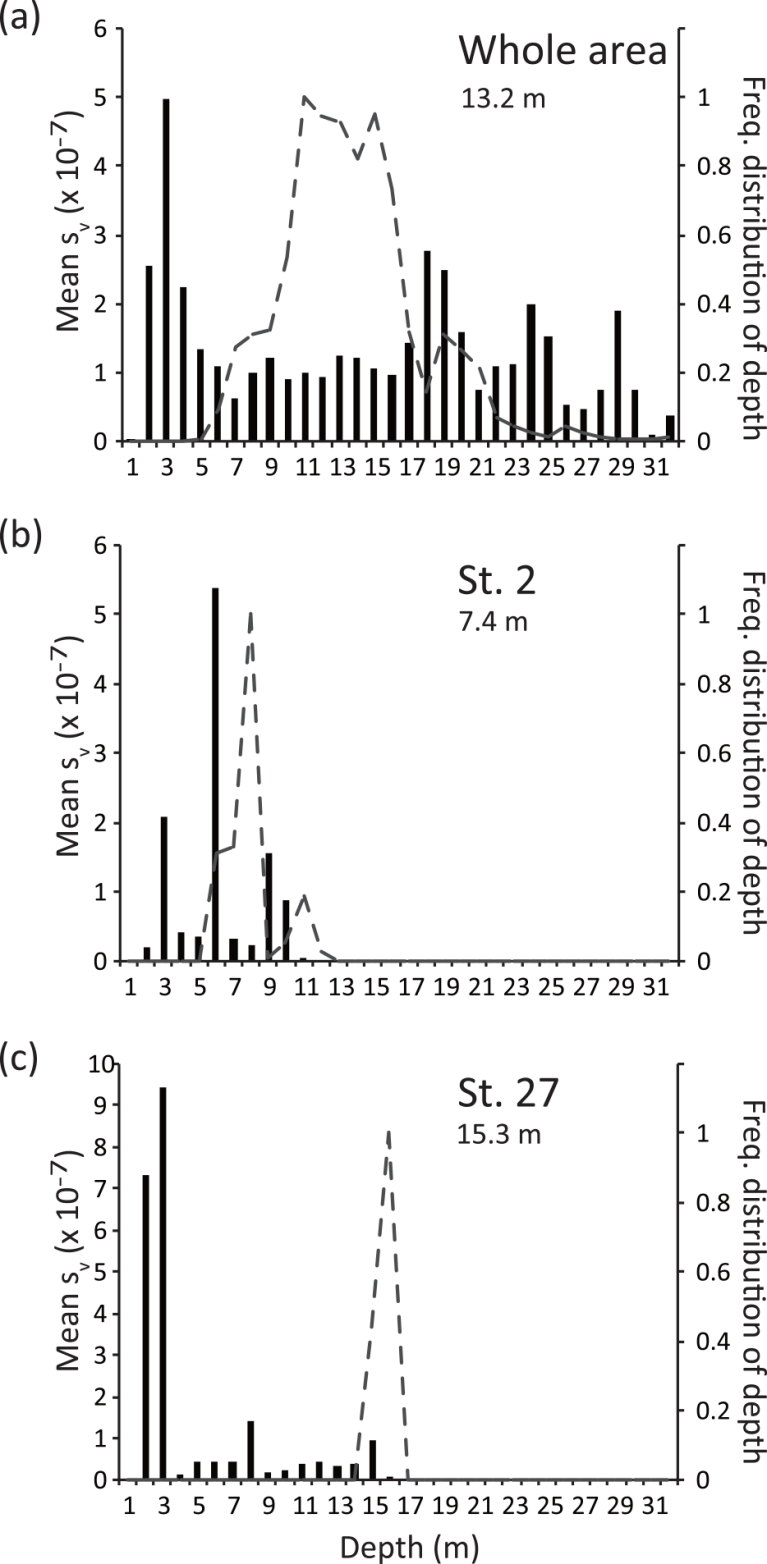
Vertical distributions of echo intensity and the sea bottom. a, data from all research areas; b, data within 150 m of St. 2; c, data within 150 m of St. 27. Bars indicate the mean echo intensity (the average of s_v_) of each depth interval. Dashed lines indicate frequency distribution of water depth. Number below station names indicates the mean water depth.

The s_v_ values obtained along the cruise trajectory varied horizontally although a meaningful geographic trend was not apparent (Fig 3). On the other hand, echo intensity was generally higher near the surface than near the bottom (Fig 4). Unlike eDNA concentration, echo intensity was high near the sea bottom around St. 2 and it was the highest near the surface around St. 27.

### Association between eDNA concentration and fish biomass

The water column fraction that best explained the obtained eDNA concentration differed when analyzing all data compared to the analyses of data excluding outliers; for the former, water columns of 10 m in radius horizontally and the entire water column vertically (i.e., from surface to bottom) were selected for both surface and bottom water samples, whereas for the latter, water columns of 150 m in radius horizontally and 5 m vertically were selected for surface samples and water columns of 50 m in radius horizontally and 2 m vertically were selected for bottom samples (Table 1; S3 Table; see S4 Table for AIC values of the best 10 models). Note that the following results and discussion are based on analyses using the echo intensity of the selected water column sizes. All covariates (echo, depth, filter, inv-dist and no-echo) and some interactions among covariates were included in the model with minimal AIC (Table 1).

**Table 1:** Selected explanatory variables, selected size of water column, and estimated partial regression coefficient of ‘echo’ for each filter series.

		All data	Data except outliers
Selected explanatory variables^a^		inv-dist	inv-dist
		depth	depth
		filter	filter
		echo	no-echo
		no-echo	filter:no-echo
		inv-dist:filter	inv-dist:filter
		filter:echo	depth:filter
		filter:no-echo	depth:echo
			inv-dist:depth:filter
			depth:filter:echo
Size of water column	Surface Horizontal (radius)	10 m	150 m
	Surface vertical	Entire column	5 m
	Bottom Horizontal (radius)	10 m	50 m
	Bottom vertical	Entire column	2 m
Estimated coefficient of echo^b^^,^^c^	Surface filter series 1	4.967 (1.958, 7.976)	7.556 (5.592, 9.519)
	Surface filter series 2	1.952 (-1.057, 4.961)	3.397 (1.433, 5.361)
	Surface filter series 3	0.691 (-2.318, 3.700)	1.439 (-0.524, 3.403)
	Bottom filter series 1	4.967 (1.958, 7.976)	-0.881 (-2.263, 0.501)
	Bottom filter series 2	1.952 (-1.057, 4.961)	-0.167 (-1.549, 1.216)
	Bottom filter series 3	0.691 (-2.318, 3.700)	0.118 (-1.265, 1.500)
	*R*^2^	0.521	0.665

^a^ See Materials and Methods section for full model.

^b^ Coefficient values of the selected explanatory variables are shown in S3 Table.

^c^ The 95% confidence intervals, estimated using the delta method, are presented in parentheses.

**Fig 5:**
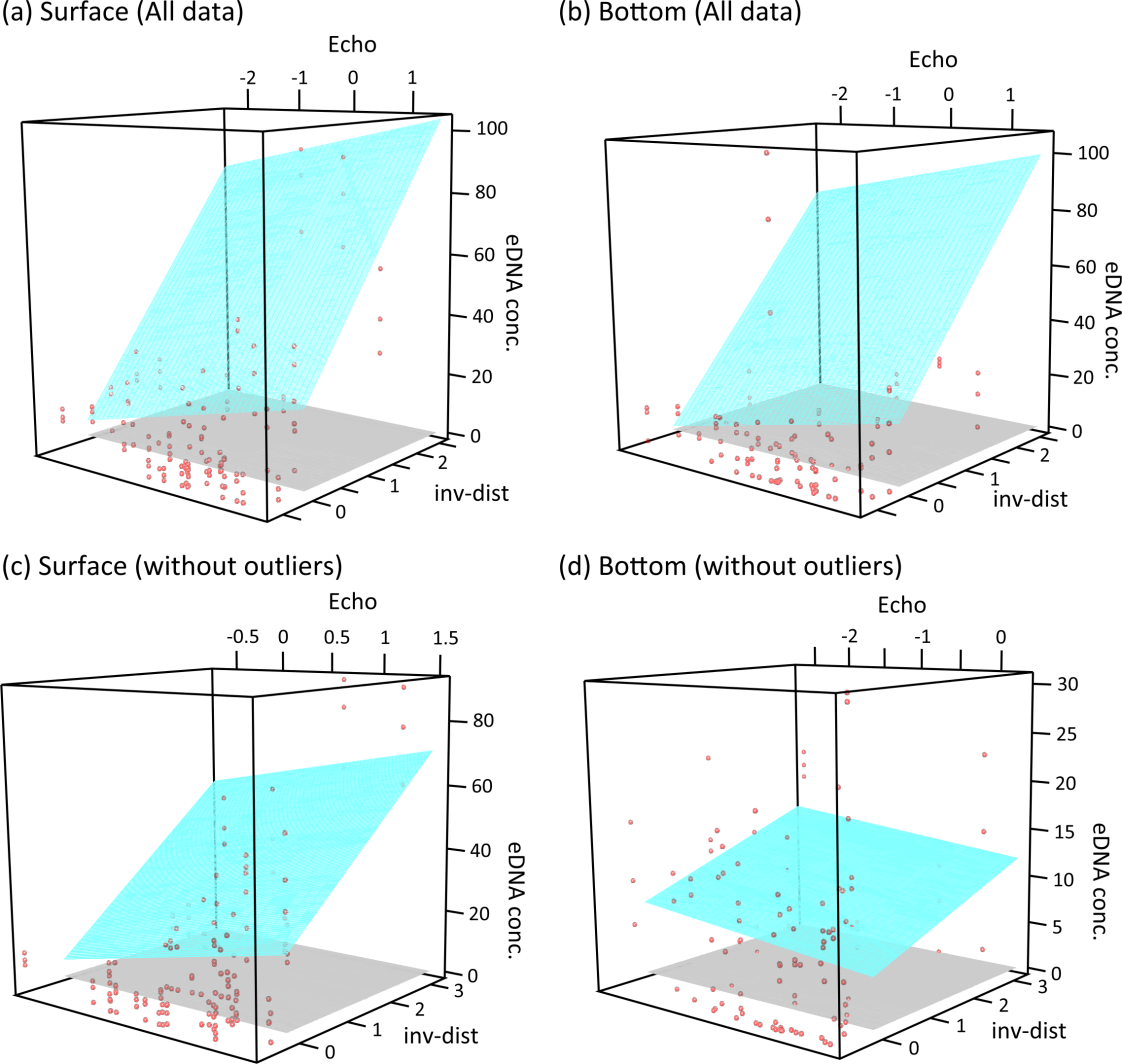
Regression surface. Relationships among eDNA concentrations (only results from filter series 1 are shown), local echo intensity (‘echo’ in regression analysis) and a measure of the inverse of distance between sampling stations and the fish market (‘inv-dist’ in regression analysis). Regression surfaces (blue), which were assessed using linear regression analysis, are indicated. Upper panels show the results of all data (a, surface water; b, bottom water) and lower panels show the results of data without outliers (c, surface water; d, bottom water).

Statistically significant partial associations between the estimated eDNA concentration and echo intensity were observed (Table 1; Fig 5). In the analysis of the entire dataset, a statistically significant positive relationship was observed in the surface and bottom water of filter series 1. On the other hand, in the analyses of the dataset without outliers, a statistically significant positive relationship was found in the surface water of filter series 1 and 2, while a partial association between echo intensity and eDNA concentration was not evident in the bottom samples.

Some other covariates were also significantly associated with eDNA concentration. We especially note the positive relationship between inv-dist, the distance index between the fish market and sampling sites, and eDNA concentration, which indicates that eDNA concentration tended to be high at sites that were located near the market (Fig 5). Moreover, inv-dist (coefficient was 30.264) had a greater coefficient than ‘echo’ (coefficient was 4.967; see S3 Table).

Unlike surface samples, bottom samples frequently shows the lower bound of eDNA concentration (i.e., copy number of eDNA = 0). Analysis based on linear regression models sometimes underestimate an association between explanatory and dependent variables when data includes a high proportion of the lower bound value (i.e., zero inflated dataset). To account for this, we also applied the Tobit regression model, which can accommodate censored dependent variables. As a result, we ensured that quantitatively similar results were obtained by fitting the Tobit regression model (S3 Table).

## Discussion

### Association between eDNA concentration and fish biomass in west Maizuru Bay

We found significant partial associations between spatial variation in estimated eDNA concentration from jack mackerel and echo intensity. This suggests that the concentration of eDNA reflects the fish distribution and biomass across west Maizuru Bay (Table 1; S1 Table). Although the association between eDNA and echo intensity was slightly different among filter series, eDNA concentrations from all filter series were positively correlated with estimated fish biomass, especially when considering all data (Table 1). Positive associations between density of target organisms and eDNA concentration have been indicated by relatively small-scale systems (e.g., mesocosm experiments [23,35], field research [18,19,24]). West Maizuru Bay (~11 km^2^) is larger and the amount of flow should be greater than such previous research systems. Although water flow in the bay would homogenize eDNA, our results suggest that eDNA concentration indicates biomass of marine fish.

On the other hand, the present study suggests that eDNA sources other than live individuals may confound distribution and biomass estimations using the eDNA method. For example, the concentration near the wholesale fish market (St. 2) was much higher than at the other sampling stations and variation in eDNA concentration among sites were strongly associated with distance from the fish market (S3 Table), such that the eDNA method exhibited a different spatial distribution of jack mackerel than the acoustic survey method (Figs 2 and 3). Because the fish market should be the only major source of jack mackerel eDNA (herein referred to as “exogenous eDNA”) in west Maizuru Bay, we were able to evaluate a partial correlation between eDNA concentration and echo intensity by statistical models including the distance from the fish market as an explanatory variable. However, when there is more than one source of such noise, a deliberate sampling design and analysis would be required to estimate spatial distribution and biomass using the eDNA method. In addition to predictable sources of exogenous eDNA such as fish markets, dead bodies would be a significant source of eDNA if they are trapped in a small area (e.g., structures on the sea bottom such as dips and rocks). The high eDNA concentration in the bottom water of St. 27 might have been due to such a cryptic source of exogenous eDNA. Thus, estimation of fish biomass using eDNA method is not simple. However, by controlling effects of exogenous DNA, this method is potentially useful to estimate the biomass and distribution of fish resources in seas. In addition, the eDNA method would be more efficient in waters isolated from human activities, for example alpine lakes and oceanic areas.

### Spatial scale of the association between eDNA and fish biomass

The relevant sizes of water columns selected by AIC were relatively small (10–150 m in radius; S4 Table), although we examined water columns with radii up to 250 m. Moreover, models with a water column of 250 m radius showed larger AIC values than models with water columns of other sizes. These results suggest that eDNA concentration would reflect jack mackerel biomass within 150 m of each sampling station (Table 1). The spatial range in which eDNA concentration reflects the target fish biomass would depend on the degradation, advection and diffusion (i.e., concentration homogenization) of eDNA. eDNA degrades over time [36,37] and therefore its concentration should decrease with distance from individual sources [38]. Previous studies have suggested that eDNA from marine fish may decrease more slowly than that of freshwater fish [13,39,40]. If jack mackerel eDNA degrades as slow as some other marine fish species (1.5–4.6% reduction per hour [40]), we might detect eDNA released 1 or 2 days prior to sampling. On the other hand, water flow would homogenize eDNA concentrations and decrease detectability of eDNA using PCR. In a stream with a water flow rate > 10 L/s, eDNA is diffused and its concentration is homogenized within 30 m from an eDNA source [41]. Although water flow was not measured in this study (but Sato et al. [42] reported that the current speed in west Maizuru Bay is a few cm/s), eDNA would be diffused immediately at our research site. Even if jack mackerel eDNA persists in the environment, the diffusion effect by water flow will reduce old eDNA. The concentration at each sampling station, therefore, is likely to reflect jack mackerel biomass at the time of water sample collection within 10–150 m from the sampling station. The remarkable similarity of such spatial scale was suggested by the eDNA-based research conducted at a different field site, Monterey Bay Kelp Forest [43] despite different methodologies and different target taxa. Considering this similarity, our estimation of spatial scale of eDNA is very convincing. However, it should be taken into consideration that the best water column size differed among datasets, and even for the same dataset some uncertainty still remained (S4 Table). Furthermore, acoustic survey cannot detect all the jack mackerels in the bay because the echo sounder detect only schools and individuals just below research vessel. This might also affect our results. Therefore, different type of study is needed to confirm horizontal spatial scale of eDNA. A caged fish experiment [41] would be helpful for understanding how large an area can be covered by a single water sample.

**Fig 6:**
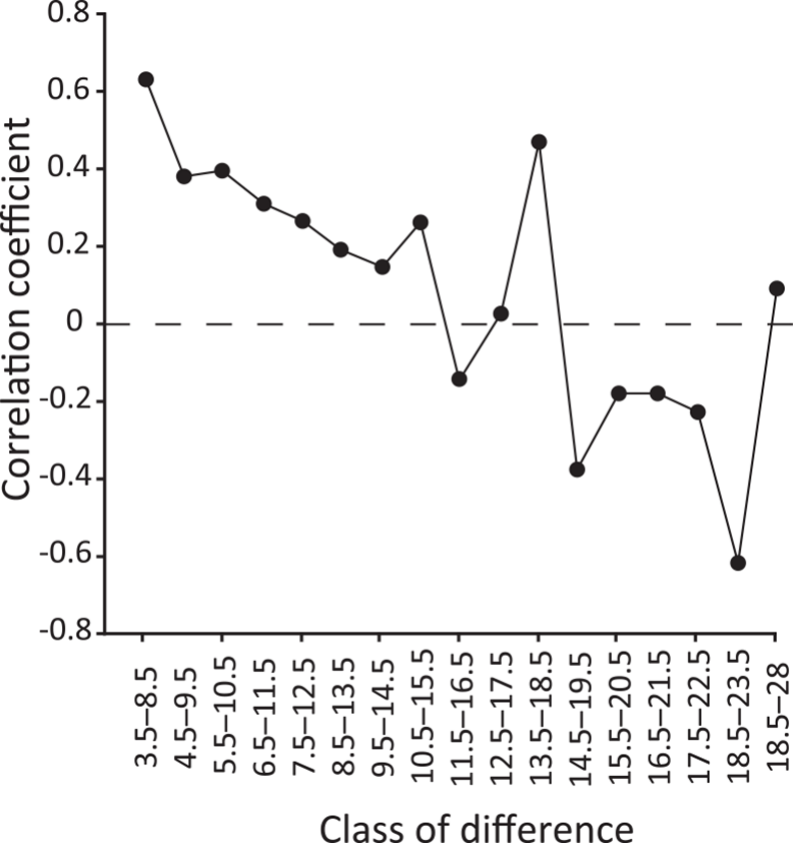
Correlation coefficient between eDNA concentration in surface and bottom waters. Y-axis indicates vertical distance between surface- and bottom-sampling positions, i.e., the coefficient value of the class of 3.5–8.5 m was calculated using samples obtained at stations where water depth is 3.5–8.5 m. Correlation coefficient was relatively high when using samples from shallower stations, while it was low when using samples from deeper stations. This figure was depicted based on eDNA concentrations of filter series 1.

The vertical spread of eDNA might also be minor in west Maizuru Bay. The best vertical sizes differed for the total eDNA dataset compared to eDNA data without outliers (i.e., both surface and bottom at St. 2 and bottom at St. 27); the entire water column (from surface to bottom) was selected with the minimal AIC for all data, whereas small sizes (2 or 5 m) were selected for data without outliers (Table 1). At St. 27, the eDNA concentration in the bottom water was higher than in the surface, whereas the opposite was true for echo intensity (Fig 4). Similarly, eDNA and echo intensity showed different vertical patterns at St. 2; the eDNA concentration was very high in both surface and bottom water but echo intensity was high near the bottom. The much higher eDNA concentrations of the outliers compared to the other samples and the incongruent pattern between eDNA results and the acoustic survey would affect the results (e.g., biomass was biased toward sea bottom while eDNA concentration is much higher in surface sample than in bottom sample for St. 2 and opposite pattern for St. 27). Moreover, the eDNA concentrations of the surface samples were not correlated with those of bottom samples when the outliers were excluded, suggesting limited transfer of eDNA between surface and bottom water. In addition, the correlation coefficient between eDNA concentration in surface and bottom waters tended to decline with an increase in vertical distance between the surface and bottom at sampling stations (Fig 6). These results correspond with the fact that vertical movement of seawater becomes weak in mid June due to stratification caused by the increasing temperature of the surface water (Sawada *et al*. unpublished data). Thus, our results might suggest limited vertical spread of eDNA.

### Advantages of quantitative analysis of eDNA

To evaluate spatial variation in eDNA concentration, we used a quantitative analysis of eDNA instead of DNA metabarcoding analysis. Quantitative analysis has an advantage in that the obtained results, that is, eDNA concentrations, can be compared among studies. This advantage also allows comparisons of temporal variation in eDNA concentration, thereby facilitating the study of temporal trends or fluctuations in eDNA concentration (e.g., [21,44,45]). By continuing water collection in Maizuru Bay, fluctuation in jack mackerel biomass can be monitored. Such information would be of substantial use for management of this species. For example, it might be useful to evaluate a relationship between migration season of the species and environmental factors. On the other hand, quantitative analysis using species-specific primers and probes does not provide information about co-occurring species. Metabarcoding analysis combined with fish-universal primers and high-throughput sequencing technology would reveal the fish community in a water sample [46]; from fish community data, species interactions might be inferred. Thus, metabarcoding analysis of the collected water samples in the present study may possibly reveal whether the spatial distribution of jack mackerel is affected by other fish species.

### Quick surveillance using the eDNA method and future tasks for accurate estimations of distribution and biomass using eDNA

Our findings suggest that the eDNA method will allow for quick surveillance in seas and large lakes. We collected water samples at 47 stations in west Maizuru Bay (~11 km^2^) in approximately 6 hours. This implies that a snapshot of localized fish resources over a large area can be revealed using the eDNA method combined with a grid survey such as we conducted in the present study. Several studies on stream organisms that conducted surveillance over large areas successfully found rare or threatened species using the eDNA method [19,21,45,47]. On the other hand, for accurate measurements of biomass based on eDNA concentration, several problems need to be solved; our results suggest that exogenous DNA complicates an association between eDNA and fish biomass. Inspection for potential exogenous DNA sources is needed prior to field research, and such exogenous DNA sources should be considered in sampling design and statistical analysis. In addition, a direct relationship between eDNA concentration and the biomass of the target species is also needed for accurate measurements of biomass [23,24,35]. A difference in eDNA release rates between developmental stages or body sizes [40] would have to be considered. Such basic knowledge about release, degradation and diffusion of eDNA under field conditions is required. If this method provides more accurate estimations of biomass, it will be useful for various issues in marine fish resources, for example, finding localized fish resources (e.g., localized spawning site) in a large area, cost-effective long-term monitoring of biomass fluctuation, and screening sea areas where threatened fish occur.

## Supporting Information

S1 Fig A summary of calculations of local echo intensity. Local echo intensity was determined as the mean of s_v_ values (gray points) within a water column multiplied by water area. (a) Buffer area is defined as a circle with a radius of assigned distance from a sampling station. Buffer area is usually identical with water area of a column although when there were land areas within the buffer area, actual water area (green) is a circle area without the overlapping land area (dark gray). (b) Green areas indicate the vertical range of a water column. We obtained s_v_ values that reflect fish biomass in a cube 1 m on a side using quantitative echo sounder. The integrated s_v_ is the sum of s_v_ values beneath each data point in assigned vertical range.
(EPS)

S1 File Data matrix and R scripts for regression analyses (executable in R). (ZIP)

S1 Table Average copy number of jack mackerel eDNA in a 2 μL template DNA solution and local echo intensity at each sampling station. (XLSX)

S2 Table Variance inflation factor. (XLSX)

S3 Table Coefficient of linear regression models (a) and Tobit regression models (b) that are selected by AIC.
(XLSX)

S4 Table AIC values of the best ten models. (XLSX)

S1 Text Evaluation of carryover through sampling devices. (DOCX)
