# Correction: Environmental DNA as a 'Snapshot' of Fish Distribution: A Case Study of Japanese Jack Mackerel in Maizuru Bay, Sea of Japan

**DOI:** 10.1371/journal.pone.0153291

**Published:** 2016-04-04

**Authors:** Satoshi Yamamoto, Kenji Minami, Keiichi Fukaya, Kohji Takahashi, Hideki Sawada, Hiroaki Murakami, Satsuki Tsuji, Hiroki Hashizume, Shou Kubonaga, Tomoya Horiuchi, Masamichi Hongo, Jo Nishida, Yuta Okugawa, Ayaka Fujiwara, Miho Fukuda, Shunsuke Hidaka, Keita W. Suzuki, Masaki Miya, Hitoshi Araki, Hiroki Yamanaka, Atsushi Maruyama, Kazushi Miyashita, Reiji Masuda, Toshifumi Minamoto, Michio Kondoh

The images for Figs 2 and 3 are incorrectly switched. The image that appears as Fig 2 should be Fig 3, and the image that appears as Fig 3 should be Fig 2. The figure captions appear in the correct order.

**Fig 2:**
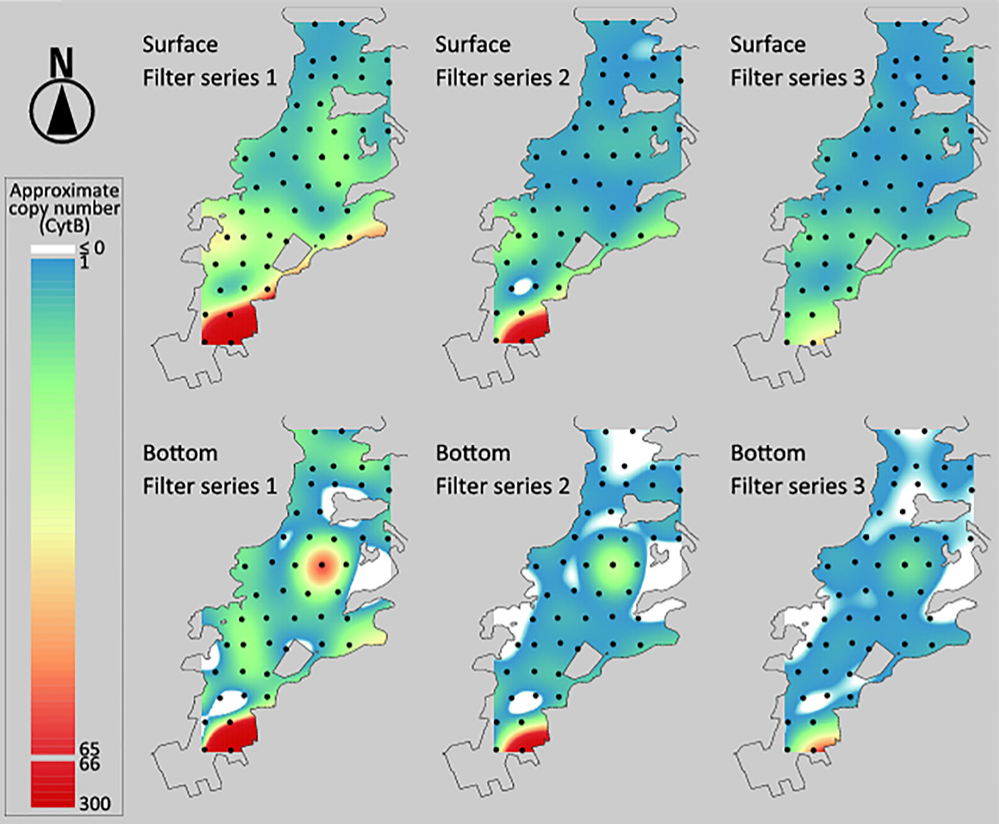
Spatial approximation of jack mackerel eDNA concentration. Based on CytB gene copy number in a 2 μL template DNA solution at the 47 sampling station, spatial variation of jack mackerel eDNA in west Maizuru Bay was estimated by approximation. The level of the approximate eDNA concentration is indicated by colors between red (relatively high concentration) and blue (low concentration). White areas suggest that the concentration approximated using a regularized spline is ≤ 0. Spatial approximation was performed using a regularized spline with a tension parameter of 40.

**Fig 3:**
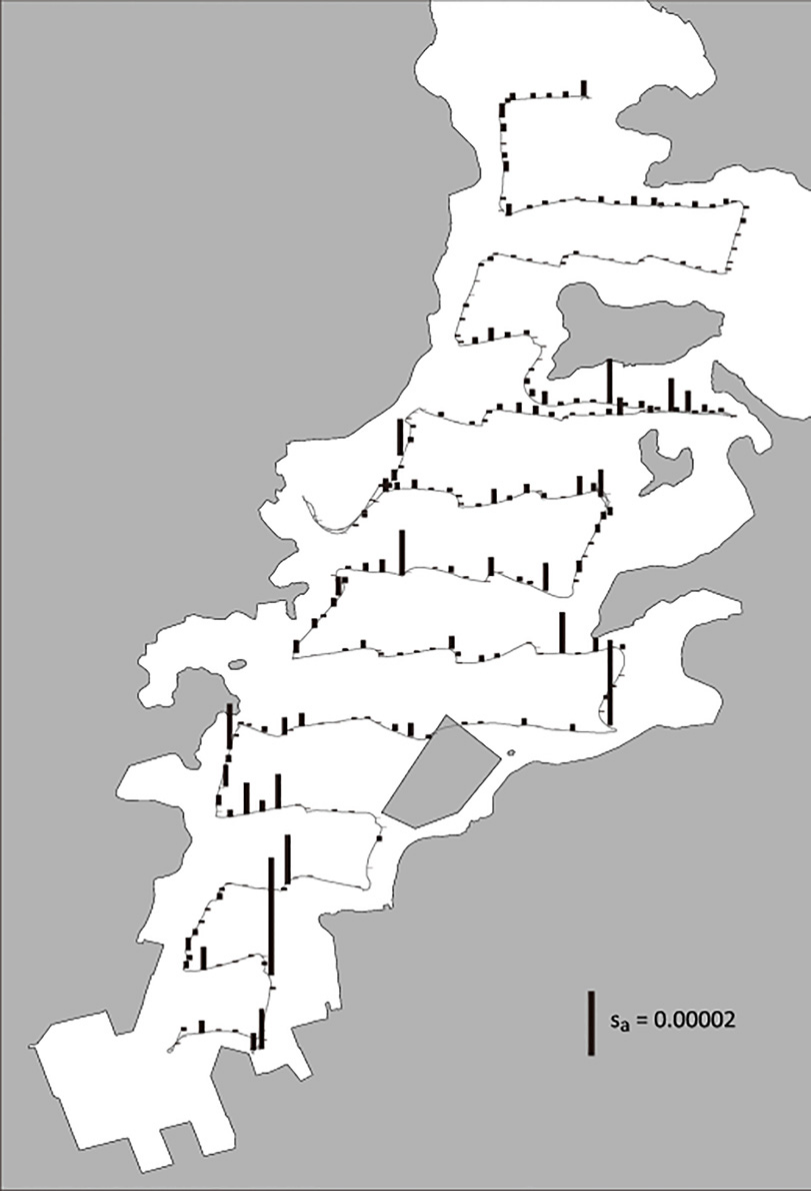
Observed fish biomass using echo sounder. Vertical bar on the cruise track (gray line) indicates local s_a_ values (i.e., fish biomass observed using quantitative echo sounder), which is the integrated s_v_ of a water column with a cross-sectional area of 1 m^2^. This figure is depicted according to s_a_ extracted every 80-m intervals. Note that this figure shows a summary of field observation using echo sounder. We used s_v_ values rather than s_a_ values as index of fish biomass in regression analyses (see S1 Fig).
