# Supplementary material for: Environmental DNA as a ‘Snapshot’ of Fish Distribution: A Case Study of Japanese Jack Mackerel in Maizuru Bay, Sea of Japan
Source: PLoS One. 2016 Mar 2;11(3):e0149786. doi: 10.1371/journal.pone.0149786 (PMC4775019; doi:10.1371/journal.pone.0149786)
Supplement: S1 Text — (DOCX) [file pone.0149786.s007.docx]

**S1 Text: Evaluation of carryover through sampling devices**

Yamamoto et al.
Environmental DNA as a ‘snapshot’ of fish distribution: A case study of Japanese jack mackerel in Maizuru Bay, Sea of Japan

Because bucket and van Dorn sampler that were used for water sampling was not bleached in our research on 18 June 2014, we conducted the additional experiment on 29 September 2015 to evaluate whether serious carryover of eDNA between sampling stations occurred. At five sampling stations (St. 1, 2, 5, 11 and 22), we collected a 1 L seawater in the way described in main text (see Materials and Methods) using the sampler soaked in high concentration eDNA solution (called as “es” sample), in which jack mackerels had been kept for three days. Then, we bleached the samplers and collected a 1 L seawater again without soaking in the eDNA solution (called as control sample). The samplers were bleached until the next sampling event. The collected seawater was filtered in the same way and the obtained filters were kept in freezer. The filtration equipment was bleached after every filtration and equipment negative controls (artificial seawater) were obtained at every station. Thus, we obtained five filters at every station (two bucket samples, two van Dorn samples and a negative control). After eDNA extraction, eDNA concentration of those filters were quantified using qPCR. Difference in eDNA concentration between es and control samples were examined by paired *t* test using R.

As a result, the concentration of the prepared eDNA solution was 65.585 copies in a 2 µL template solution just before the first sampling (i.e. St. 1). Given that the range of eDNA concentrations of seawater were between 1.320 and 12.082 copies in a 2 µL template solution, the prepared eDNA solution had enough higher concentration than seawater. eDNA concentrations of seawater samples collected using the samplers (i.e. bucket and van Dorn sampler) soaked in high concentration eDNA solution were not statistically different from those of samples collected using bleached samplers (paired t test, *t* = 0.1491; *p* = 0.889 for bucket and *t* = 2.015; *p* = 0.114 for van Dorn sampler). Because this additional experiment finished within as little as two hours, the eDNA solution would keep higher concentration than seawater during the experiment.
